# Coexistence of Antiferromagnetism and Superconductivity in Heavy Fermion Cerium Compound Ce_3_PdIn_11_

**DOI:** 10.1038/srep15904

**Published:** 2015-10-30

**Authors:** M. Kratochvílová, J. Prokleška, K. Uhlířová, V. Tkáč, M. Dušek, V. Sechovský, J. Custers

**Affiliations:** 1 Charles University in Prague, Faculty of Mathematics and Physics, Ke Karlovu 5, 121 16 Prague 2, Czech Republic; 2 Department of Structure Analysis, Institute of Physics of the CAS, v.v.i., Na Slovance 2, 182 21 Prague, Czech Republic

## Abstract

Many current research efforts in strongly correlated systems focus on the interplay between magnetism and superconductivity. Here we report on coexistence of both cooperative ordered states in recently discovered stoichiometric and fully inversion symmetric heavy fermion compound Ce_3_PdIn_11_ at ambient pressure. Thermodynamic and transport measurements reveal two successive magnetic transitions at *T*_1_ = 1.67 K and *T*_N_ = 1.53 K into antiferromagnetic type of ordered states. Below *T*_c_ = 0.42 K the compound enters a superconducting state. The large initial slope of *dB*_c2_/*dT* ≈ – 8.6 T/K indicates that heavy quasiparticles form the Cooper pairs. The origin of the two magnetic transitions and the coexistence of magnetism and superconductivity is briefly discussed in the context of the coexistence of the two inequivalent Ce-sublattices in the unit cell of Ce_3_PdIn_11_ with different Kondo couplings to the conduction electrons.

The vast majority of cerium intermetallic compounds investigated to date have been compounds which have only one crystallographic site for the Ce-ion. As such, the physical properties of these dense Kondo lattices are determined by the competition between the magnetic inter-site interaction, i.e., the indirect RKKY exchange interaction, and the demagnetizing on-site Kondo interaction. This competition is well described by the Doniach diagram^1^; the ground state depends on the relative value of the respective RKKY and Kondo energies, 
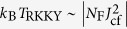
 and 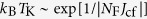, with *N*_F_ being the conduction band density of states at the Fermi level and *J*_cf_ the coupling constant (hybridization) between 4*f* (Ce) and conduction electrons. For small values of 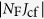 the compound’s ground state is magnetic while, for high values of 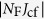, the Kondo effect dominates and the ground state is nonmagnetic. The value of *J*_cf_ itself depends critically on the local environment of the 4*f* electron. This aspect plays a crucial role in compounds exhibiting inequivalent crystallographic Ce-sites per unit cell constituting different Ce–sublattices. The different local symmetry of each inequivalent Ce-ion results in a different hybridization strength and hence each corresponding Ce–sublattice is characterized by its own Kondo temperature. Therefore, it gives the possibility of having different ground states for each sublattice. Examples are for instance Ce_7_Ni_3_, Ce_5_Ni_6_In_11_ and Ce_3_Pd_20_Si_6_. The first-mentioned compound crystallizes in the hexagonal Th_7_Fe_3_ structure in which the Ce-ions reside in three inequivalent sites: (i) one atom in sublattice Ce1 (Wyckoff site 2*b*: 3 Ni and 12 Ce neighbors); (ii) three in sublattice Ce2 (6*c*: 4 Ni, 11 Ce) and (iii) three in sublattice Ce3 (6*c*: 4 Ni, 11 Ce but different distances than for Ce2). From thermodynamic and magnetic studies it was suggested that at low-*T* the Ce1 ions are responsible for antiferromagnetic (AFM) order at *T*_N_ = 1.8 K, the Ce2 ions are associated with heavy fermion (HF) behavior (*T*_K_ ≈ 4 K) and the Ce3 ones give rise to intermediate valence contributions^2^. The second compound, Ce_5_Ni_6_In_11_, has a base centered orthorhombic unit cell. There are ten Ce-ions per unit cell. They occupy two different Ce-sites. Eight Ce-ions sit at Wyckoff position 8*p* (Ce1) while the remaining two occupy the 2*c* site (Ce2). The compound has been classified as an intermediate HF system with *γ* ≈ 145 mJ/(mol_Ce_ · K^2^) and exhibits two successive AFM magnetic phase transitions at *T*_N2_ = 1.10 K and *T*_N1_ = 0.63 K which have been attributed to the Ce1 and Ce2 sublattices, respectively^3^. The analysis of the entropy indicated a weak interaction between the two magnetic sublattices below *T*_N2_. The latter compound, Ce_3_Pd_20_Si_6_, has recently attracted much interest because of its unusual quantum critical behavior^4^. The compound crystallizes in the cubic structure of space group 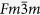. It harbors two different crystallographic Ce-sites, both with cubic symmetry but one with coordination 16 (tetrahedral 8*c*: all Pd) and one with coordination 18 (octahedral 4*a*: 12 Pd, 6 Si). At *T* = 0.5 K, Ce_3_Pd_20_Si_6_ undergoes a quadrupolar phase transition and at *T*_N_ = 0.31 K a transition in an AFM ordered state^5^. Each ordered state has been associated with a particular sublattice, meaning that the quadrupolar state and the dipolar coexist at low temperatures.

Yet, the field of Kondo lattice compounds with multiple distinct local moments (4*f* (Ce)) per unit cell is still uncharted terrain. This despite the fact that interplay between different ground states, which in these compounds might come from interaction between sublattices with different electronic or magnetic order, often give rise to new interesting phenomena. Indeed recent theoretical work of Benlarga *et al.* examines the question of a Kondo lattice with two distinct Kondo ions^6^. Their model describes a unit cell with two inequivalent Ce-sites. Each experiences different hybridization 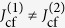 which reflects in different (sublattice) Kondo temperatures *T*_K1_≠*T*_K2_, respectively. For simplicity of the discussion it was assumed *T*_K1_>*T*_K2_ 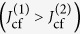. Long-range magnetic ordering was induced by allowing for Ce1-Ce2 interaction and a schematic phase diagram has been constructed. Particular interesting becomes the situation *T*_K1_>*T*>*T*_K2_ where one enters a region in the phase diagram of so-called partial Kondo screening. Partial refers to the fact that only 4*f* (Ce1) moments are effectively screened by Kondo effect forming heavy particle states. Hence, as has been pointed out by the authors, any magnetic phase in this regime will manifest characteristics of a weakly polarized HF phase coexisting with properties of typical local-moment magnetism originating from the strongly polarized and little screened 4*f* (Ce2)-moments. The compound Ce_7_Ni_3_ mentioned earlier shows exactly such behavior. Although not explicitly discussed, in a broader sense one can speculate that under certain conditions the HF sublattice becomes superconducting while the second sublattice remains magnetically ordered.

In this work we present first low-temperature magnetization, specific heat and resistivity measurements on the recently^7^,^8^ discovered HF system Ce_3_PdIn_11_. The compound belongs to the Ce_*n*_*T*_*m*_In_3*n*+2*m*_ class of layered materials which comprises a numerous amount of compounds including CeCoIn_5_, CeRhIn_5_ and Ce_2_RhIn_8_ 9,10,11. Ce_3_PdIn_11_ can be regarded as “intermediate step” between the cubic CeIn_3_ which orders antiferromagnetically at *T*_N_ = 10.1 K^12^ and tetragonal Ce_2_PdIn_8_ being a HF superconductor with *T*_c_ between 0.45 and 0.7 K depending on the crystal quality^13^,^14^,^15^,^16^. It crystallizes in the typical tetragonal structure (space group *P*4/*mmm*) based on the AuPt_3_-type (CeIn_3_-block) and PtHg_2_-type (*T*in_2_) units alternating along the [001]-direction as depicted in the Fig. 1. The unit cell possesses two inequivalent crystallographic Ce-sites denoted by Ce1 and Ce2. What distinguishes Ce_3_PdIn_11_ from previously investigated multi-site compounds is that the Ce1–Ce1 nearest-neighbor distance is exactly equal to the Ce2–Ce2 nearest-neighbor distance. That means, the distance as a parameter to explain the various behaviors of the sublattices like in other compounds plays no role^17^. Instead, the ground state properties of the sublattices are solely determined by the respective hybridization of the Ce-ions in our compound. A second point which outrivals Ce_3_PdIn_11_ from previous multi-site Ce-compounds is that the local environment of both Ce-ions are not uniquely related to Ce_3_PdIn_11_ but already exists in closely related compounds. As displayed in Fig. 1, Ce2 resides in the 1*a* Wyckoff site (*C*_4*v*_ symmetry) featuring CeIn_3_ environment, whereas the Ce1-site occupies the 2*g* position (*D*_4*h*_ symmetry). Its surrounding atoms are identical with those of the Ce-ions in Ce_2_PdIn_8_. Preliminary results on Ce_3_PdIn_11_ revealed two successive magnetic transitions at *T*_1_ ≈ 1.7 K and *T*_N_ ≈ 1.5 K and a pronounced enhancement of the Sommerfeld coefficient^8^. Our extended low-*T* experiments presented here show that below *T*_c_ = 0.42 K the compound enters a HF superconducting state, suggesting coexistence of long-range magnetic order and HF superconductivity in Ce_3_PdIn_11_ at ambient pressure.

## Results

**Figure 2:**
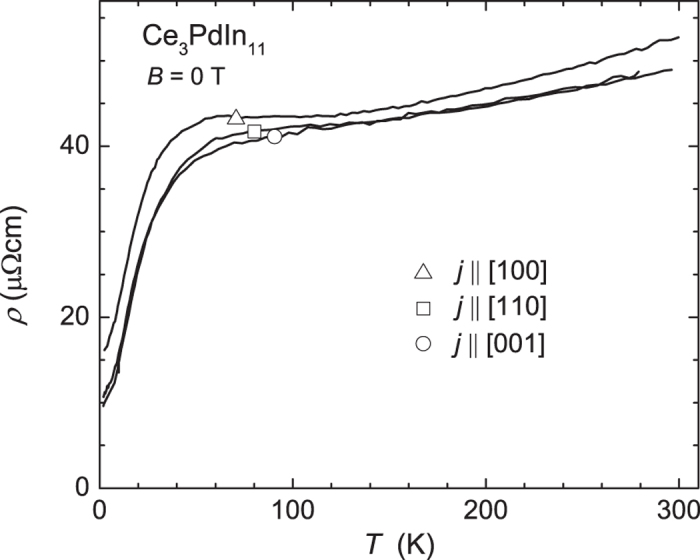
The electrical resistivity *ρ*(*T*) of Ce_3_PdIn_11_ with current *j* || [100] (triangles), ||[110] (open squares) and ||[001] (circles) from room temperature down to 2 K. The residual resistivity ratio *RRR* = *ρ*_300K_/*ρ*_2K_ equals 5.

The electrical resistivity *ρ*(*T*) of Ce_3_PdIn_11_ with current applied along different crystallographic axes is shown in Fig. 2. At high temperatures above 80 K the resistivity *ρ*(*T*) is weakly temperature dependent with *dρ*/*dT*>0, passes through a shallow maximum at *T*_max_ ≈ 60 K (*j* ‖ [100]) with lowering temperature, and then rapidly decreases signaling the formation of coherent Bloch states at low temperatures. At *T* = 2 *K* the resistivity reaches *ρ*(2 K) ~ 16 *μ*Ω cm, 11 *μ*Ω cm and 10 *μ*Ω cm for *j* ‖ [100], ‖[001] and ‖[011], respectively. The overall behavior is similar to that reported from a previous study performed on polycrystalline material^7^. The room temperature resistivity value corresponds well to ours, i.e., *ρ* = 55 *μ*Ω cm has been reported compared to 52 *μ*Ω cm measured for *j* ‖ [100] measured in our study. The maximum structure at *T*_max_ is at 30 K and more pronounced in the polycrystal. The low temperature resistivity value, however, differs significantly being worse for the polycrystal (*ρ*(2 K) ≈ 34 *μ*Ω cm) which is an indication that our single crystal is of higher quality. From our single crystal data we observe that the resistivity is fairly isotropic despite the complex tetragonal structure.

**Figure 3:**
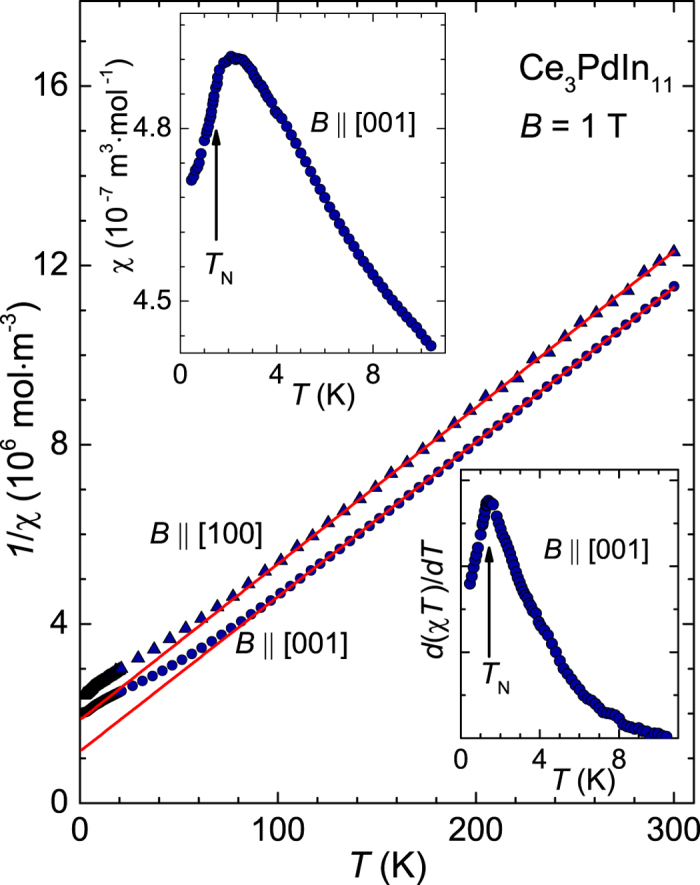
Temperature dependence of the inverse of the susceptibilities 1/*χ*(*T*) of Ce_3_PdIn_11_ for the [100] (triangles) and [001] (circles) directions in an applied field of *B* = 1 T. The solid (red) lines are Curie-Weiss fits to the data. The upper left inset shows the low temperature susceptibility for *B* ‖ [001] in more detail. The lower right inset displays the corresponding derivative *d*(*χT*)/*dT*.

The magnetic susceptibility *χ*(*T*) displays similar isotropic behavior. At *T* = 4 K the ratio between parallel and perpendicular [001]-axis yields *χ*_[001]_/*χ*_[100]_ = 1.2. This suggests a strong influence of the cubic CeIn_3_ structure unit on the magnetic correlations in this compound. In Fig. 3 we plotted the inverse susceptibility 1/*χ* = *B*/*M* as function of temperature along both crystallographic directions in applied field of *B* = 1 T. For temperatures *T* >85 K and *B* ‖ [100] and for *T* >100 K and *B* ‖ [001] the susceptibility follows Curie-Weiss law. As inferred from Fig. 3 the data 1/*χ*(*T*) for both directions are parallel resulting in an equal effective moment of *μ*_eff_ = 2.43 *μ*_B_ per Ce-ion. The value is close to the Hund’s rule value of 2.54 *μ*_B_ for a free Ce^3+^ ion. In addition, because of the nearly magnetocrystalline isotropy of Ce_3_PdIn_11_ the extrapolated Weiss temperatures are close, yielding 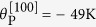 and 
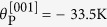
. The upper inset of Fig. 3 displays the low-temperature susceptibility for *B* ‖ [001] in greater detail. Below about 6 K *χ*(*T*) gradually deviates from the high-temperature behavior and exhibits a broad hump with a maximum at *T* = 2.4 K. In fact, Ce-based compounds (*J* = 5/2) commonly exhibit a low-*T* maximum in *χ*(*T*). This is expected from the theory of orbitally degenerate Kondo impurities^18^. However, we have to account for the crystal electric field (CEF) level scheme. In tetragonal crystal symmetry the *J* = 5/2 line splits into three doublets, 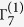, 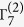 and Γ_6_. Assuming that the Ce1-ion in Ce_3_PdIn_11_ has similar CEF scheme as Ce in Ce_2_PdIn_8_, it follows that the first excited level is located at about Δ_CEF_ ≈ 60 K^13^. The situation for Ce2-ion is more unclear. It exhibits tetragonal symmetry contrary to cubic symmetry of the Ce-ion in CeIn_3_. The first excited CEF level is at Δ_CEF_ ≈ 140 K^19^. In a rough approximation we may assume a similar level splitting of the *J* = 5/2 line for Ce2. Because the Kondo temperature in Ce_2_PdIn_8_ and CeIn_3_ is much smaller than the CEF splitting being of the order of *T*_K_ ≈ 10 K^13^,^19^ only the ground state doublet is relevant and the effective degeneracy of the moments becomes *J* = 1/2. In that case the Kondo effect produces a maximum in susceptibility at *T* = 0. We therefore attribute the maximum structure to the presence of short-range AFM correlations and determine the transition into long-range AFM ordered state in accordance with work of Fisher^20^: for a mean-field transition, the dc susceptibility should show a cusp at *T*_N_, whereas for classical critical behavior the derivative *d*(*χT*)/*dT* should diverge at *T*_N_ with a specific exponent (i.e., 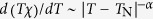) so that *T*_N_ occurs at an inflection point below a rounded maximum. From specific heat measurements two inflection points in *χ* at *T*_1_ = 1.67 K and *T*_N_ = 1.53 K can be expected^8^. We observe only one at approximately 1.45 ± 0.1 K as displayed in the lower inset of Fig. 3. The value coincides well with the lower specific heat jump at *T*_N_. The upper *T*_1_-anomaly seems not to be reflect in *χ*(*T*) (*B* ‖ [001]) but it is not excluded that it might be visible for perpendicular direction. Alternatively, the *T*_1_–signature is too small, and beyond resolution of our current measurement setup.

**Figure 4:**
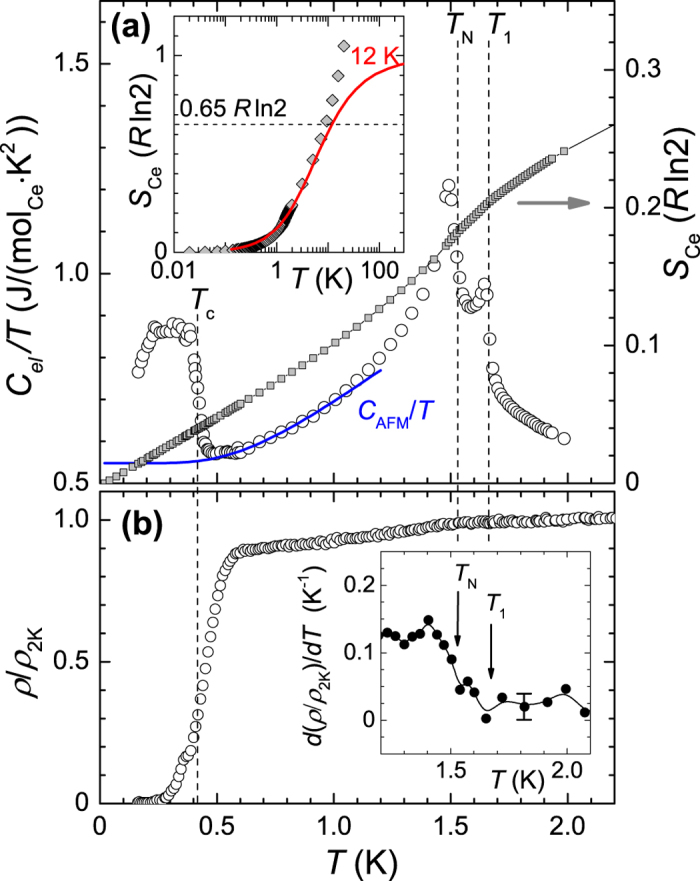
Zero field low-temperature results on Ce_3_PdIn_11_. (**a**) the specific heat *C*_el_/*T* vs. *T* (open symbols) in units of J/(mol_Ce_ · K^2^). The transitions are clearly visible at *T*_1_ = 1.67 K, *T*_N_ = 1.53 K and *T*_c_ = 0.42 K. The solid line represents the function 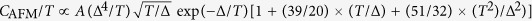 with values for *A* and Δ given in the text. The right ordinate displays the presumable magnetic entropy *S*_Ce_ per mol_Ce_ in units of *R*ln2. The inset compares *S*_Ce_ with the spin-1/2 Kondo model of Desgranges and Schotte with *T*_K_ = 12 K (red curve)^30^. (**b**) The temperature dependence of the electrical resistivity normalized to 2 K. The current was applied *j* ⊥ [001]. Inset: temperature derivative of *ρ*/*ρ*_2K_ in the temperature region around the magnetic transitions. The error bar indicates scattering of the data points.

We continued our investigation down to lower temperatures by means of specific heat (*C*/*T*) and resistivity experiments. Measurements were performed on the same single crystal to avoid sample dependent effects. In order to trace the associated anomalies and to complement the methods among each other the results of *C*(*T*)/*T* and *ρ*(*T*) in zero field are displayed one below the other in Fig. 4. The synthesis of the respective non-magnetic La and Y–compounds was unsuccessful so far. In order to retrieve the electronic part of the specific heat, *C*_el_/*T*, we approximated the specific heat data in the interval 10<*T*<25 K by *C*(*T*)/*T* = *C*_phon_/*T* + *γ*, where the Debye expression, 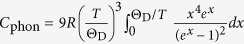, accounts for the phononic and the Sommerfeld term (*γ*) for the the electronic contribution. The fit yields a characteristic temperature of Θ_D_ ~ 182 K and *γ* = 0.890 J/(mol · K^2^). *C*_phon_/*T* was subtracted from the total specific heat data and we obtained *C*_el_/*T* presented in Fig. 4a. In our treatment we have disregarded the contribution arising from CEF. A more accurate analysis including this term is not possible because of the limited fitting interval and because of the fact that the exact CEF level scheme for Ce_3_PdIn_11_ is unknown. The CEF contribution, *C*_CEF_/*T*, is somehow captured by *C*_phon_/*T* and *γ*. To give an estimate about *C*_CEF_/*T* we might take the CEF scheme of Ce_2_PdIn_8_ for comparison. The first excited level (Δ_CEF_ ≈ 60 K) gives rise to a Schottky-type of anomaly with a maximum at around 25 K (*C*_CEF_/*T* ≈ 0.17 J/(mol_Ce_ · K^2^))^14^. Below it falls off exponentially and at 10 K reaches a value of *C*_CEF_/*T* ≈ 0.05 J/(mol_Ce_ · K^2^). We can assume that similar values are reached for Ce_3_PdIn_11_. This means, that our simplified fitting of the heat capacity data leads to an overestimation of the phononic and electronic contribution due to the inclusion of the CEF contribution. Towards lower temperatures, because *C*_CEF_/*T* and *C*_phon_/*T* become small, the error in *C*_el_/*T* displayed in Fig. 4 becomes negligible. Figure 4a shows three distinct features. Upon cooling down a first anomaly appears at *T*_1_ = 1.67 K which is immediately followed up by a second one at 1.53 K, being marked as *T*_N_ and visible by a *λ*-like structure in the *C*_el_/*T* data. Like in susceptibility, the higher transition *T*_1_ is not observed in *ρ*(*T*) within the accuracy of the measurement. *T*_N_ is noticeable (Fig. 4b). It is manifested by a subtle change of the slope as shown more clearly by the temperature derivative *d*(*ρ*/*ρ*_2K_)/*dT* (inset of Fig. 4b). More intriguing is the third feature appearing in *C*_el_/*T* at approximately 0.4 K. The jump marks the entrance into a superconducting phase, as it is evident from the corresponding resistivity data. The transition is fairly broaden which makes a proper analysis impossible. It resembles the first results on UPt_3_, where after improving sample quality it turned out that the broad specific heat jump actually consists of two very close superconducting jumps^21^. A more likely explanation is that the broadening results from an artifact caused by a strong increase of *C*_el_/*T* of a normal state background. Consequently the expected decrease becomes compensated. Such an effect can be observed for instance in CeIr(In_1−*x*_Cd_*x*_)_5_ 22. To extract some numbers for comparison, we define the SC transition temperature being at half of the jump height, *T*_c_ = 0.42 K, which corresponds roughly with the onset of a second step in the resistivity data. The measured specific heat discontinuity, Δ*C* divided by the linear term in the specific heat at the transition (*γT*_c_) equals 0.73 (*γ* = 0.55 ± 0.03 J/(mol_Ce_ · K^2^)). This value is well below that expected from BCS theory (Δ*C*/*γT*_c_ = 1.43). However, such downsized number is not unusual when the transition is particularly broad, see for instance ref. 23,24.

Finally, we estimated the magnetic entropy 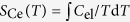 by cutting off the superconducting transition and extrapolating the low-temperature tail of the *T*_N_–transition towards zero. For this we employed a fit expression accounting for the cooperative effect of spin-wave excitations^25^, with *A* = 0.0109 J/(mol · K^2^) per Ce and Δ = 2.74 K (solid line in Fig. 4a). It is fair to state that a second-order mean-field BCS-type of fit^26^ gives similar result due to the limited fitting range of 0.5–1.2 K. *S*_Ce_(*T*) is plotted as closed symbols in Fig. 4a. The entropy deliberated at *T*_1_ is only 0.2*R*ln2 per mol_Ce_. This is well below the value (*R*ln2) associated with the lifting of the degeneracy of the ground state doublet suggesting magnetic ordering with substantially reduced magnetic moments.

### B – T Magnetic Phase Diagram

**Figure 5:**
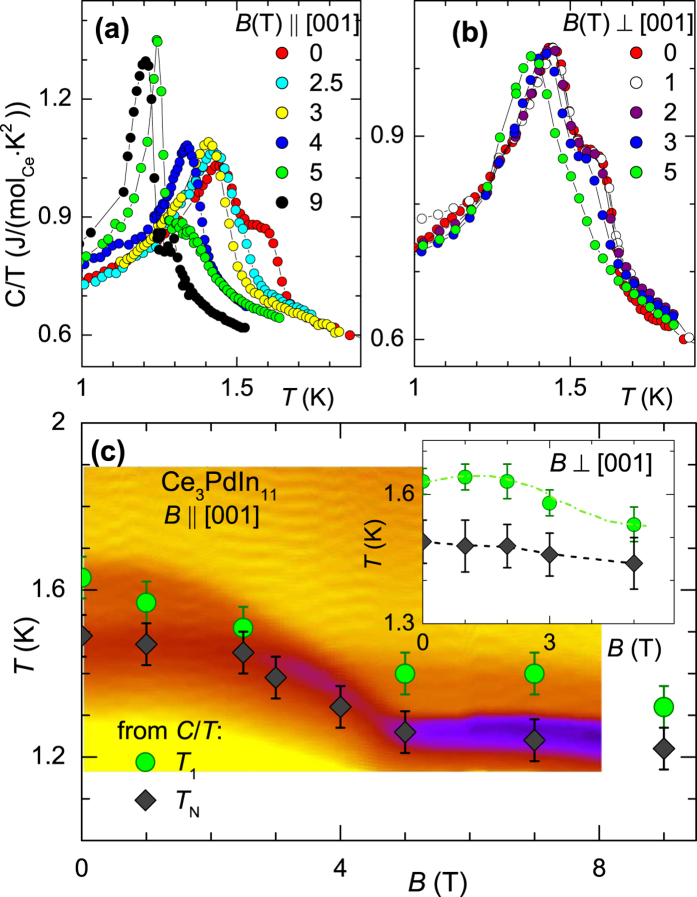
Specific heat as function of temperature for various values of applied magnetic fields (**a**) *B* || [001] and (**b**) *B* ⊥ [001]. The temperature – magnetic field phase diagram (**c**) for *B* ‖ [001] mapped out by the thermal response technique (see text). The green circles and black diamonds show *T*_1_ and *T*_N_, respectively, as obtained from the data present in (**a**). Inset depicts the *B* − *T* phase diagram for *B* ⊥ [001] axis.

The influence of an applied magnetic field on the magnetic transitions was studied by means of specific heat experiments. The magnetic transitions are most pronounced in this measurement. The subtraction of the phonon and CEF terms has been omitted because we are only interested in the change of the transition temperatures. Experiments were conducted on a sample from a different batch than the one shown in Fig. 4. Figure 5 cumulates the results. We first focus on the behavior for *B* ‖ [001]. From Fig. 5a it can be seen that in small fields *B* < 2.5 T the transition *T*_1_ shifts to lower temperatures while *T*_N_ remains almost unaffected. At *B* ≈ 3 T both transitions seem to merge. In higher fields *B* >4 T we observe again two transitions. Whether or not these two transitions indeed correspond to the low-field transitions as suggested by the figure is subject of further investigation. Our attention was devoted to the measurements in *B* = 5 T and above. In this field region the transition at *T*_N_ shows a sharp *λ*-like shape, typical for a first-order type transition. Such increase in sharpening and peak size has also been found for the field-induced transition in CeRhIn_5_ 11.

Figure 5b shows the specific heat for applied field perpendicular to [001]-axis. The *T*_1_-transition and the successive ordering at *T*_N_ are clearly observed in low fields. *T*_1_ first shows a tiny increase, likely caused by a realignment of the moments, before gradually decreasing with increasing field. The lower transition temperature remains almost unchanged for *B* <5 T and moves monotonically to lower temperatures in larger fields. The constructed *B* − *T* phase diagrams are presented Fig. 5c. The phase diagram for *B* ‖ [001] has been measured in more detail by the method of analyzing the response of the compound to thermal heat pulses. By this method a heat pulse resulting in a Δ*T* = 1 K temperature change of the sample (mounted quasi-adiabatically) to the bath temperature *T*_bath_ was applied. The subsequent thermalization of the sample temperature to a temperature *T* was monitored over time *t*. The color plot shows the derivative *d*(Δ*T*)/*dt* as a function of the bath temperature and field (yellow: small change in *dT*/*dt*; blue/violet: large change in *dT*/*dt*). The thermal relaxation result is complemented by data points of *T*_1_ and *T*_N_ obtained from specific heat experiments (*T*_1_: circles, *T*_N_: diamonds). The inset displays the respective phase diagram for *B* ⊥ [001].

From Fig. 5c it becomes more evident that the two transitions, *T*_1_ and *T*_N_, merge at ≈3 T. Moreover, in fields >4 T the *T*_N_ anomaly sharpens as inferred by the rapid change in color code. Such a behavior indicative of a pronounced first-order type of transition and hence corroborates our observation already made in *C*/*T*. An intriguing aspect is that the sensitivity of the magnetic transitions with respect to the applied field is weakest for *B* ⊥ [001] and strongest for *B* ‖ [001]. For the two other magnetically ordered compounds of the Ce_*n*_*T*_*m*_In_3*n*+2*m*_ class of materials, CeRhIn_5_ and Ce_2_RhIn_8_, it is vice versa^11^.

### The Superconducting State

**Figure 6:**
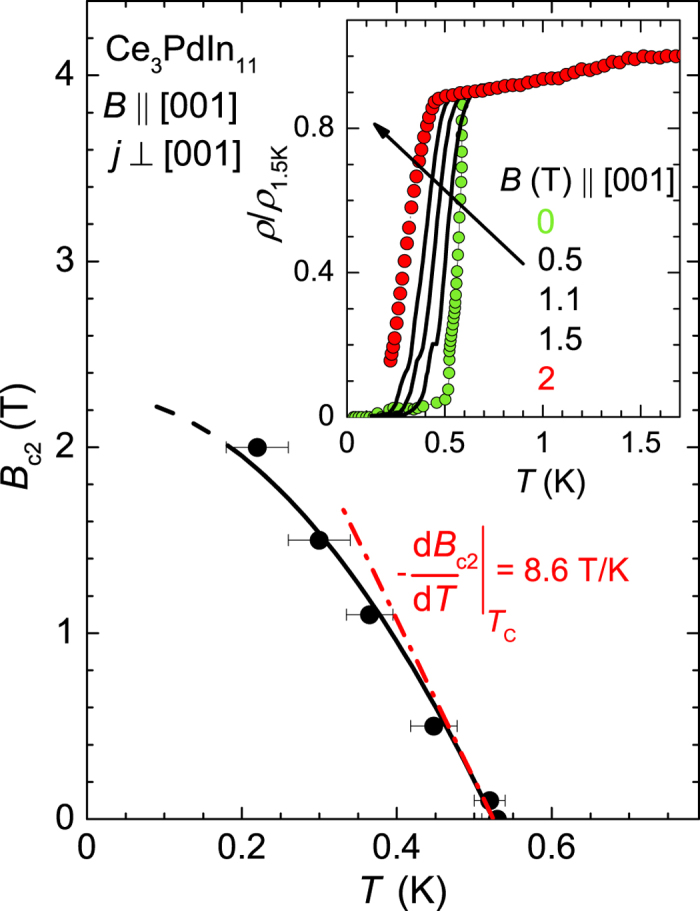
Temperature dependence of the upper critical field of *B*_c2_ as derived from resistivity experiments on Ce_3_PdIn_11_. Typical curves are shown in the inset. Current was *j* ⊥ [001] and the field was applied parallel to the [001]-axis. The red solid line yields –*dB*_c2_/*dT* = 8.6 T/K.

The observation of ambient pressure bulk superconductivity below *T*_N_ gives Ce_3_PdIn_11_ a unique place within the Ce_*n*_*T*_*m*_In_3*n*+2*m*_ family. So far pressure or doping was necessary to induce SC in a magnetically ordered compound. Corresponding examples are CeRhIn_5_, Ce_2_RhIn_8_, Ce(Rh_1−*x*_Ir_*x*_)In_5_ or CeCoIn_5_ doped with Sn and Cd^22^,^27^,^28^. Figure 6 shows the superconducting phase diagram of Ce_3_PdIn_11_ for *B* ‖ [001] and *j* ‖ [100]. The jump in resistivity in various external fields is plotted in the inset of Fig. 6. The data are normalized to the value at 1.5 K (*ρ*(*T*)/*ρ*(1.5 K)). We defined the second step in the resistivity drop as *T*_c_ as discussed earlier. Within the accuracy of the measurement, the value of *T*_c_ fairly well corresponds with that obtained from the transition temperature in the specific heat. As expected, the application of magnetic fields reduces *T*_c_, giving rise to a rather large change of the initial slope of the upper critical field *dB*_c2_/*dT* ≈ −8.6 T/K. Extrapolation of the field dependent transition temperatures to *T* → 0 using a simple mean-field type expression *B*_c2_(*T*) = *B*_c2_(0)[1 − (*T*/*T*_c_)^2^] yields *B*_c2_(0) ≈ 2.3 T.

## Discussion

Our experimental results show that Ce_3_PdIn_11_ undergoes two successive magnetic transitions into antiferromagnetic-type ordered states at *T*_1_ = 1.67 K and *T*_N_ = 1.53 K. At lower temperatures the compound becomes superconducting (*T*_c_ = 0.42 K). Beyond that the data reveal that the magnetic ground state exhibits a reduced magnetic moment and suggest a heavy fermion superconducting state. Hence, Ce_3_PdIn_11_ takes a unique place being the first known full inversion symmetrical stoichiometric Ce-based compound which shows coexistence of magnetic order and superconductivity at ambient pressure. As we mentioned in the introduction the existence of two inequivalent Ce-sites in Kondo lattice systems can lead to the observation of unusual ground states and complex phase diagrams (e. g., ref. 2, 3, 4). Such an expectation found some general justification in the framework of the Kondo lattice model describing the consequences of the coexistence of two inequivalent local moment Ce sublattices on the ground state properties, when linked to different Kondo couplings to the conduction electrons^6^. Since Ce_3_PdIn_11_ belongs to this class of Kondo systems, we will try in the following to provide an explanation of the novel phenomena in the context of two Ce sublattices.

The tetragonal structure of Ce_3_PdIn_11_ comprises two crystallographically inequivalent Ce-sublattices. The Ce-ions on the Ce1-sublattice (2 ions per f.u.) exhibit a Ce_2_PdIn_8_ environment and the Ce-ions on the Ce2-sublattice (1 ion per f.u.) possess a CeIn_3_ surrounding. Due to different local environments, each sublattice will have its individual CEF scheme and Kondo scale (*T*_K1_ and *T*_K2_). As a result complex interacting mechanisms are expected in Ce_3_PdIn_11_. In a first approach we can gain some insight by a quantitative analysis of *C*(*T*)/*T* and *S*(*T*). As shown in Fig. 4 the electronic specific heat coefficient *γ* of Ce_3_PdIn_11_ is found to be 0.55 ± 0.03 J/(mol_Ce_ · K^2^) when extrapolated to zero temperature. This large *γ* places Ce_3_PdIn_11_ into the heavy fermion category. However, since there are two kinds of Ce-ions (Ce1 and Ce2) with different site symmetries and neighboring ions it is quite likely that the two do not contribute equally to the density of states at the Fermi level, i.e., *γ* = 0.55 J/(mol_Ce_ · K^2^) is an average value. The same argument accounts for the magnetic entropy *S*_Ce_ which is depicted in the same figure. The average *S*_Ce_ per mol of cerium deliberated at *T*_1_ equals 0.2*R*ln2, that is one-fifth of the value expected for a ground state doublet. Thus there is a considerable reduction of the magnetic entropy. We relate the deficiency of *S*_Ce_ (*T*_1_) to the presence of the Kondo effect which partially lifts the twofold degeneracy above *T*_1_. In such a case it can be shown that *S*_Ce_(*T*_1_) = *S*_K_(*T*_1_/*T*_K_), where *S*_K_(*T*_1_/*T*_K_) is the Kondo entropy at *T*_1_ 29. The relation holds if the first exited level 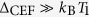 and 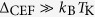. From earlier discussion of the temperature dependence of the susceptibility we concluded that Δ_CEF_ ≈ 60 K. Thus the former condition is satisfied in Ce_3_PdIn_11_. Typical Kondo scales in the Ce_*n*_*T*_*m*_In_3*n*+2*m*_ class of materials are in the range between 2 and 10 K suggesting that also the second condition is fulfilled^10^. Further, using the Bethe ansatz for spin-1/2 Kondo model, Desgranges and Schotte calculated the specific heat and the corresponding magnetic entropy^30^. By inserting the experimentally found *S*_Ce_ into the above given relation we obtain the ratio *T*_1_/*T*_K_. The estimated Kondo temperature for Ce_3_PdIn_11_ hence yields 12 K. This Kondo temperature is an average of *T*_K1_ and *T*_K2_. The value agrees well with the estimated Kondo temperature from the relation 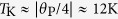
31. The inset in Fig. 4a compares *S*_Ce_ with the calculated values of the spin-1/2 Kondo model^30^. We observe that below *T*_1_ the experimental data fall under the theoretical curve as expected (entropy is absorbed in the transition). For 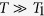 the *S*_Ce_ values exceed the calculated ones which is an indication for the presence of additional contributions to the specific heat, most likely originating from excited CEF levels which have not been subtracted from the *C*/*T* data.

The small entropy release of ~0.2*R*ln2 at *T*_1_ implies a strong screening of the 4*f*-moment which gives rise to a small ordered moment in the magnetic state. This is consistent with the observation of a small signal in *χ*(*T*) at the magnetic transition temperatures. The nature of these two phase transitions at *T*_1_ and *T*_N_ needs to be studied further in particular with regard to the two Ce-sites. We notice that with an increasing field parallel to [001] both *T*_1_ and *T*_N_ decrease which is consistent with the fact that both phase transitions are of antiferromagnetic type. It is thus expected that the magnetic moments of both Ce–sublattices are mutually interacting (RKKY-type interaction). The long-range magnetic interaction on the other hand competes with the Kondo interaction. In Ce_3_PdIn_11_ *T*_K_ will be in turn determined by the effective strength and type of interplay of the two sublattice Kondo temperatures *T*_K1_ and *T*_K2_ 6. The complexity of the interactions may account for the intriguing magnetic phase diagram of Ce_3_PdIn_11_ being distinct different from the other two magnetically ordered compounds of the Ce_*n*_*T*_*m*_In_3*n*+2*m*_ family (CeRhIn_5_ and Ce_2_RhIn_8_) which have only one Ce-site per f.u.^11^.

Next we briefly discuss the superconducting state. A remarkable close analogy is found between the SC properties of Ce_3_PdIn_11_ and Ce_2_PdIn_8_. The SC transition temperature of our compound is *T*_c_ = 0.42 K while for Ce_2_PdIn_8_ values between 0.45 and 0.7 K have been reported^13^,^14^,^15^,^16^,^32^. In addition, the upper critical field yields *B*_c2_ = 2.3 T for Ce_3_PdIn_11_ and 2.5 T for Ce_2_PdIn_8_ which is very similar^32^. Difference is solely observed in the initial slope of *B*_c2_ being *dB*_c2_/*dT* ≈ −8.6 T/K and *dB*_c2_/*dT* ≈ −13.5 T/K, respectively.

Before we discuss such a similarity to Ce_2_PdIn_8_ and to avoid confusion we want to exclude the possibility of having Ce_2_PdIn_8_ impurity phase in our samples. As we recently reported, our single crystal Ce_3_PdIn_11_ samples have been carefully analyzed by X-ray diffraction and Scanning Electron Microscopy with an Energy Dispersive X-ray detector (see Methods section) and clearly excludes the presence of any Ce_2_PdIn_8_ inclusions.

The large observed SC discontinuity of Δ*C*/*T*_c_ ≈ 0.89 J/(mol · K^2^) in Ce_3_PdIn_11_ corresponds to a SC jump Δ*C*/(*γT*_c_) = 0.73 in Ce_3_PdIn_11_ and amounts to 51% of the BCS-value. Such a high ratio suggests that a large portion of the sample is in the superconducting state. One possibility to understand the observed similarities of the superconducting properties could be the fact that Ce_3_PdIn_11_ has a layer structure and the Ce1-site in Ce_3_PdIn_11_ exhibits a Ce_2_PdIn_8_ environment (see above). However this needs further microscopic proof.

In summary, at ambient pressure Ce_3_PdIn_11_ undergoes magnetic transitions into an AFM state at *T*_1_ = 1.67 K and an order-to-order transition into a second AFM state at *T*_N_ = 1.53 K. The entropy analysis shows an magnetic entropy release at *T*_1_ of only 0.2*R*ln2 indicating that the ordered moment is small. The strong reduction of the magnetic moment is due to Kondo effect. Below *T*_c_ = 0.42 K the compound becomes superconducting. The size of the jump in specific heat and the large value of initial slope *dB*_c2_/*dT* ≈ −8.6 T/K indicate that the Cooper pairs are formed of heavy quasiparticles. In this sense Ce_3_PdIn_11_ is unique being the first known full inversion symmetrical stoichiometric heavy fermion material based on Ce exhibiting coexistence of both attributes, magnetism and superconductivity at ambient pressure. In fact, the crystal structure of Ce_3_PdIn_11_ possesses two inequivalent Ce-sites. The Ce1-site exhibits Ce_2_PdIn_8_ surrounding and the Ce2-site has CeIn_3_-like environment. Each Ce-sublattice is expected to have a different Kondo temperature and different RKKY interaction within the sublattice. The interplay between these two sublattices in Ce_3_PdIn_11_ is likely strong and gives rise the complex ground state. To unravel and to gain deeper insight in the particular mechanisms at work, microscopic methods are indispensable. For instance ^105^Pd and ^115^In NQR and NMR can provide information about the magnetic moment on the Ce-ion but also about the superconducting gap structure which is an important quantity to reveal the mechanism behind Cooper-pairing. On the other hand neutron experiments can resolve the magnetic structure. Finally we feel that our experimental results would stimulate further experimental and theoretical efforts to explore the nature of electronic correlation and their theoretical understanding in this class of compounds.

## Methods

### Single crystal growth

Plate-like single crystals with mass up to ≈1 mg were grown by indium self-flux method^8^. High-purity starting materials (Ce 99.9%, Pd 99.995% and In 99.999%) in the ratio 3:25:50 were placed in high purity alumina crucibles and sealed under vacuum in quartz glass tubes together with another crucible filled with quartz wool plug for later flux removal. The crucibles were heated up to a temperature of 750 °C for several hours (8–10 h) and slowly (3 °C/h) cooled down to 550 °C. At this temperature, the material was decanted to separate the single crystals from the remaining In-flux.

The quality of the single crystals was checked by X-ray Laue diffraction in reflection geometry. The chemical composition was verified employing a Tescan Mira I LMH scanning electron microscope (SEM). The instrument is equipped with a Bruker AXS energy dispersive X-ray detector (EDX). Elemental mapping has confirmed composition homogeneity^8^. Several pieces were pulverized and examined at room temperature by means of powder X-ray diffraction (Bruker D8 Advance diffractometer with Cu-K*α* radiation). The obtained diffraction patterns were refined by Rietveld analysis using FullProf Suite. In addition, in order to refine the structural parameters more precisely and to confirm phase homogeneity, several samples were investigated by single crystal X-ray diffraction using a X-ray diffractometer Gemini with Mo-K radiation. The crystal structure was resolved by charge flipping program Superflip and adjacent refinement was done by full-matrix least-squares based on *F*^2^ using Jana2006. Experiments were performed on samples from different batches.

### Experimental Details

The physical properties of the Ce_3_PdIn_11_ samples were attained employing standard equipment. The magnetic susceptibility was determined using a MPMS 7 T SQUID magnetometer (Quantum Design) with an applied field of 1 T. Additionally, in the temperature range between 0.5 and 4 K magnetization measurements were performed using a Hall-probe which is sensitive to the dipole field created by the sample magnetization. In order to obtain absolute values the Hall probe data were scaled in the overlapping region (2–10 K) with the data measured by the MPMS. The method is still in testing stage. Resistivity was measured utilizing standard four point a. c. technique. Low-temperature measurements up to *T* ≈ 2 K were performed in a Leiden Cryogenics MCK72 dilution refrigerator. The setup is equipped with a 9 T magnet. Measurements above 0.4 K up to room temperature were done in a Physical Property Measurement System (PPMS) from Quantum Design. The low-*T* range of the PPMS guarantees sufficient overlap of low- and high-temperatures data sets. In similar manner specific heat experiments were conducted in PPMS down to 0.45 K and were continued to lower temperatures using a Quantum Design dilution refrigerator heat capacity puck mounted to the MCK72 dilution refrigerator.

## Additional Information

**How to cite this article**: Kratochvílová, M. *et al.* Coexistence of Antiferromagnetism and Superconductivity in Heavy Fermion Cerium Compound Ce_3_PdIn_11_. *Sci. Rep.* **5**, 15904; doi: 10.1038/srep15904 (2015).

## Figures and Tables

**Figure 1:**
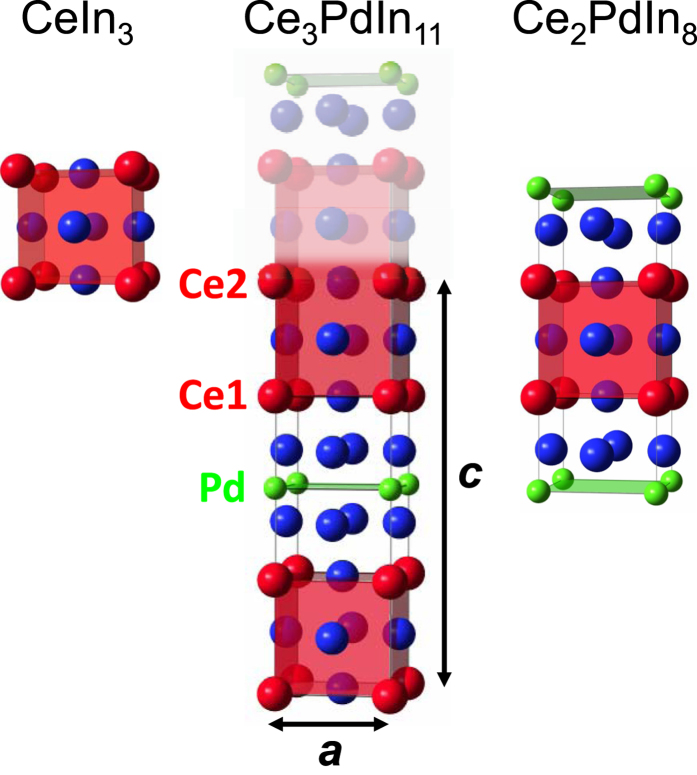
The crystal structure of Ce_3_PdIn_11_ (middle) pointing out the two inequivalent Cerium sites Ce1 and Ce2. The red shaded part emphasizes the CeIn_3_ building blocks. The lattice parameters yield *a* = 4.6896(11) Å and *c* = 16.891(3) Å. To show the local environment of the Ce2-ion more clearly, the unit cell has been extended along the [001]-direction (semi-transparent part). The unit cells of CeIn_3_ (left) and Ce_2_PdIn_8_ (right) are displayed for comparison.
